# Characteristics and outcome of burned children admitted to a pediatric intensive care unit

**DOI:** 10.5935/0103-507X.20180045

**Published:** 2018

**Authors:** Luciana Gil Barcellos, Ana Paula Pereira da Silva, Jefferson Pedro Piva, Leandra Rech, Tamires Goulart Brondani

**Affiliations:** 1 Unidade de Terapia Intensiva de Trauma Pediátrico, Hospital Municipal de Pronto Socorro de Porto Alegre - Porto Alegre (RS), Brasil.; 2 Unidade de Tratamento Intensivo Pediátrico, Hospital de Clínicas de Porto Alegre, Universidade Federal do Rio Grande do Sul - Porto Alegre (RS), Brasil.; 3 Unidade de Emergência Pediátrica, Hospital de Clínicas de Porto Alegre, Universidade Federal do Rio Grande do Sul - Porto Alegre (RS), Brasil.; 4 Serviço de Emergência e Medicina Intensiva Pediátrica, Hospital de Clínicas de Porto Alegre, Universidade Federal do Rio Grande do Sul - Porto Alegre (RS), Brasil.; 5 Departamento de Pediatria, Universidade Federal do Rio Grande do Sul - Porto Alegre (RS), Brasil.; 6 Programa de Residência em Pediatria e Terapia Intensiva, Hospital de Clínicas de Porto Alegre, Universidade Federal do Rio Grande do Sul - Porto Alegre (RS), Brasil.; 7 Unidade de Terapia Intensiva Pediátrica, Hospital Moinhos de Vento - Porto Alegre (RS), Brasil.; 8 Programa de Terapia Intensiva Pediátrica, Hospital da Criança Santo Antônio - Porto Alegre (RS), Brasil.

**Keywords:** Burns, Child, Intensive care units, pediatric

## Abstract

**Objective:**

To analyze the characteristics and outcomes of children hospitalized for burns in a pediatric trauma intensive care unit for burn patients.

**Methods:**

An observational study was conducted through the retrospective analysis of children (< 16 years) admitted to the pediatric trauma intensive care unit for burn victims between January 2013 and December 2015. Sociodemographic and clinical variables were analyzed including the causal agent, burned body surface, presence of inhalation injury, length of hospital stay and mortality.

**Results:**

The study analyzed a sum of 140 patients; 61.8% were male, with a median age of 24 months and an overall mortality of 5%. The main cause of burns was scalding (51.4%), followed by accidents involving fire (38.6%) and electric shock (6.4%). Mechanical ventilation was used in 20.7% of the cases. Associated inhalation injury presented a relative risk of 6.1 (3.5 - 10.7) of needing ventilatory support and a relative risk of mortality of 14.1 (2.9 - 68.3) compared to patients without this associated injury. A significant connection was found between burned body surface and mortality (p < 0.002), reaching 80% in patients with a burned area greater than 50%. Patients who died had a significantly higher Tobiasen Abbreviated Burn Severity Index than survivors (9.6 ± 2.2 *versus* 4.4 ± 1.1; p < 0.001). A Tobiasen Abbreviated Burn Severity Index ≥ 7 represented a relative risk of death of 68.4 (95%CI 9.1 - 513.5).

**Conclusion:**

Scalding burns are quite frequent and are associated with high morbidity. Mortality is associated with the amount of burned body surface and the presence of inhalation injury. Special emphasis should be given to accidents involving fire, reinforcing proper diagnosis and treatment of inhalation injury.

## INTRODUCTION

Burns constitute an important public health problem and represent the third leading cause of accidental death in the pediatric population.^(1)^ The most recent estimate of global burn-related mortality in children is 2,500 to 100,000.^(2)^ In Brazil, there are approximately 1 million accidents involving burns occur per year, with 100,000 patients seeking hospital care and approximately 2,500 dying directly or indirectly due to their injuries.^(3)^

The long-term prognosis of these children depends mainly on the initial approach and treatment, which can reduce mortality, the number of complications, scarring and the need for future reconstructive surgeries. The improvement in prognosis is also related to the emergence of specialized centers, advances in resuscitation protocols, individualized intensive treatment and improvements in wound dressing techniques and treatment of infection, inhalation injuries and hypermetabolism. All of these changes have had impact in the morbidity and mortality of burn patients.^(1,4)^ The mortality rate has declined globally by approximately 36% in the last decade, especially in children.^(2)^

The epidemiology of burns differs between adults and children. Most burns in children are caused by overheated liquids, followed by contact and flame burns.^(1,4)^ Scalding represents up to 85% of accidents and is more frequent in the population younger than 5 years.

The burned body surface (BBS) and the presence of inhalation injury have been associated with greater severity and a worse prognosis.^(5-7)^ A recent study in adults showed that the extent of BBS is associated with increased mortality risk, especially when the BBS is greater than 60%, rather than 40%, as was established in the previous decade.^(4,8)^

The objective of this study was to analyze the epidemiological characteristics of children hospitalized for burns in a pediatric trauma intensive care unit (ICU).

## METHODS

An observational cross-sectional epidemiological study was conducted through a retrospective analysis of hospitalization records of all burn victims younger than 16 years admitted to the pediatric trauma ICU of the *Hospital Municipal de Pronto Socorro de Porto Alegre* (HPS) from January 2013 to December 2015.

The pediatric trauma ICU of the HPS is a reference unit in the state of Rio Grande do Sul for the care of child victims of trauma and burns. It is a training unit for Pediatric and Intensive Pediatric Medicine residency programs of several institutions in the city of Porto Alegre. The unit has 8 beds, with an annual admission rate of 250 patients and an overall mortality rate of approximately 4%. The team is entirely composed of doctors certified by the *Associação de Medicina Intensiva Brasileira/Sociedade Brasileira de Pediatria/Associação Médica Brasileira* (AMIB/SBP/AMB) in the field of Pediatric Intensive Care Medicine.

The medical records of children admitted to the pediatric trauma ICU in the mentioned period with a main diagnosis of burn by any mechanism were selected. Sociodemographic variables such as age, sex and origin and clinical variables such as causal agent, BBS, presence of inhalation injury, length of hospital stay and mortality were analyzed.

In our service, we use the Lund-Browder classification to estimate BBS, taking into account areas with second- and third-degree burns. According to the body surface affected, the patients were classified into four groups: 0 - 15%, 15 - 30%, 30 - 50% and > 50% burned body surface.

Inhalation injury was characterized in children victims of fire burns who suffered injuries including burned face, nasal passages or eyelashes and carbonaceous sputum, stridor or wheezing. Due to the technical difficulties associated with pediatric patients and the availability of a bronchoscope suitable for the age group, it was not possible to perform bronchoscopy in all patients.

The Tobiasen Abbreviated Burn Severity Index (ABSI) was used as a severity score for burn patients (Table 1).^(9,10)^

**Table 1:** Tobiasen Abbreviated Burn Severity Index

Characteristics		Score
Sex		
Female		1
Male		0
Age (years)		
0 - 20		1
21 - 40		2
41 - 60		3
61 - 80		4
81 - 100		5
Inhalation injury		
Yes		1
No		0
Presence of full-thickness burn		
Yes		1
No		0
Burned body surface (%)		
1 - 10		1
11 - 20		2
21 - 30		3
31 - 40		4
41 - 50		5
51 - 60		6
61 - 70		7
71 - 80		8
81 - 90		9
91 - 100		10
**ABSI score and mortality**		
**ABSI**	**Risk of death**	**Probability of survival (%)**
2 - 3	Very low	≥ 99%
4 - 5	Moderate	98%
6 - 7	Moderately severe	80 - 90%
8 - 9	Serious	50 - 70%
10 - 11	Severe	20 - 40%
≥ 12	Maximum	≤ 10%

ABSI - Tobiasen Abbreviated Burn Severity Index.

The data were collected and tabulated in an Excel spreadsheet designed for the study. When disagreement occurred, the case was presented and discussed by the group of authors until a consensus was reached. The study was evaluated and approved by the Institution's Ethics Committee.

Continuous quantitative variables are expressed by central tendency measures (mean and median), with the respective dispersion (standard deviation or range), and groups were compared using Student's *t*-test and the Mann-Whitney U-test. Categorical variables are expressed as percentages or in descriptive form, using the chi-square test or Fisher's exact test and the relative risk for comparisons.

## RESULTS

Between January 2013 and December 2015, 671 patients were admitted to the pediatric trauma ICU of the HPS. 147 of them (20%) were children and adolescents admitted with burn as the main cause. Seven of those were excluded due to incomplete data in the medical record, and the remaining 140 patients were included in the study. Most patients were male (61.8%) and lived in the metropolitan region of Porto Alegre (70%), with a median age of 24 months and an overall mortality rate of 5% (Table 2).

**Table 2:** Characteristics of 140 patients admitted to the pediatric trauma intensive care unit for burns

Variables	Patients N = 140
Age (months)	24 (1 - 183)
Male	85 (61.8)
From the Porto Alegre metropolitan area	98 (70)
Mortality	7 (5)
Length of hospital stay (days)	8 (1 - 403)
Tracheostomy	4 (2.9)
Burned surface	12 (< 1 - 85)
Mechanical ventilation	29 (20.7)
Mechanical ventilation time (days)* (n = 29)	9 (1 - 732)
Inhalation Injury	21 (15)
Need for mechanical ventilation†	10 (41.6)

* n = 29;

† n = 21. Values expressed as medians (interquartile range) or numbers (%).

Mechanical ventilation was used in 20.7% (29/140) of the patients in the study group. Ventilatory support was required in 71.4% (15/21) of patients with associated inhalation injury and in 11.7% (14/119) of patients without inhalation injury. Associated inhalation injury presented a relative risk of 6.1 (3.5 - 10.7; p < 0.0001) of needing ventilatory support.

In the analysis of the injury mechanism (Table 3), we identified that the main cause of burns were accidents involving a heated liquid (scalding), accounting for 51.4% of hospitalizations (n = 72), followed by accidents involving fire and explosion in 38.6% (54 patients) and electric shock in 6.4% (n = 9). Five charts showed no record of the burn mechanism. When comparing the different groups according to their burn mechanism, we observed that the accident involving fire group had a higher mortality rate (11.1%) than the scalding (1.4%, p = 0.02) and electric shock (11.1%, p < 0.001) groups.

**Table 3:** Burn mechanism and associated age and mortality

Mechanism	N (%)	Age (months) Median (min-max)	Mortality N*
Scalding	72 (51.4)	15 (1 - 137)	1
Fire	45 (32.1)	78 (11 - 183)	5
Explosion	9 (6.4)	108 (8 - 146)	0
Electrical shock	9 (6.4)	47 (23 - 156)	1

Min - minimum; max - maximum.

* The burn mechanism was not associated with higher mortality (p = 0.10).

Among the 140 patients, the proportion of BBS ranged from 1% to 85%, with a median of 12%, and a length of stay in the ICU ranging from 1 to 403 days, with a median of 8 days. The overall mortality rate was 5% (Table 4). When patients were stratified according to BBS into four groups (0 - 15%, 15 - 30%, 30 - 50% and > 50%), we observed an increase in the median length of hospitalization (7, 11, 39 and 48 days, respectively) and a significant increase in mortality (0%, 2.7%, 14.3% and 80%, respectively, p < 0.002) related to a larger burned surface area.

**Table 4:** Burned surface area and associated mortality

Burned area (%)	Patients n (%)	Length of hospital stay Median (min-max)	Mortality (%)
0 - 15	84 (60)	7.0 (1 - 403)	0
15 - 30	37 (26.4)	11.0 (2 - 64)	1 (2.7)
30 - 50	14 (10)	39.0 (5 - 205)	2 (14.3)
> 50	5 (3.5)	48.0 (31-147)	4 (80.0)*

Min - minimum; max - maximum.

* Significant association between burned surface area and mortality (p < 0.002).

Twenty-one patients (all victims of accidents involving fire) presented inhalation injuries, with a mortality rate of 32.8% (p < 0.0001), which was higher than that observed the one in children with burns without lung injury (1.7%). Inhalation injury presented a relative risk of mortality of 14.1 (2.9 - 68.3) compared to patients without this associated injury.

Patients who died had a much higher ABSI than the survivors (9.6 ± 2.2 *versus* 4.4 ± 1.1, p < 0.001). We observed that patients admitted with an ABSI ≥ 7 had a mortality rate of 53.8%. An ABSi ≥ 7 was associated with a relative risk of death of 68.4 (95%CI 9.1 - 513.5).

## DISCUSSION

Burns represent the second most frequent cause of childhood accidents and are responsible for increased morbidity and functional sequelae. This relevance is evident if we consider that only in this pediatric ICU, with eight beds to treat trauma patients, over a period of 3 years, one admission due to burn occurred every 8 days, corresponding to 20% of the admissions during this period.

In Brazil, there are few specific data on the epidemiology of burns in pediatric patients. In general, the studies are observational and include adults.^(11)^

The most common injury mechanism was scalding, which usually occurs in the home environment in children younger than 5 years old.^(12)^ In an epidemiological study that reviewed the pediatric care of burn victims over 35 years (1974-2001) at Parkland Hospital in Texas, the main burn mechanism was scalding, which accounted for 42% of admissions.^(13)^ In another retrospective study that analyzed data from the National Trauma Registry of the American College of Surgeons over a period of 15 years (1995-2013), scalding occurred in 71.1% of cases, and 53% were caused by superheated liquids found in the kitchens of households, such as coffee and tea.^(14)^ These findings demonstrate the relevance of domestic accidents during a developmental stage associated with curiosity, lack of coordination and easy access to the kitchen without adequate supervision. In this sense, adequate control of the environment with an accident prevention policy would have a significant impact on this numbers.^(12,15)^

An evident association exists between the extent of BBS and mortality. A BBS greater than 30% in patients from zero to 18 years old is expected to increase the inflammatory response and, consequently, increase mortality.^(1,4,7)^ Mortality among burned patients has decreased in previous decades, and recent studies have shown a correlation between mortality and BBS > 60%.^(4,7,16,17)^ This same association was found in our study, as we observed more significant mortality in patients with a BBS > 50%.

A significant portion of deaths from fire or explosion are related to inhalation injury and the toxic effects of combustion.^(18,19)^ Recent studies suggest that between 20 and 30% of severely burned patients have associated inhalation injury, and of these, 20 - 50% require ventilatory support during the first week of treatment.^(4,5)^ Inhalation injury substantially increases mortality and, generally, endotracheal intubation is required, which increases the incidence of pneumonia. Early detection of lung injury by bronchoscopy improves survival.^(7,16,18,19)^ In the present study, mortality was strongly related to fire burns and the presence of inhalation injury.

There is no specific predictor of mortality for burned pediatric patients. The ABSI is the most commonly used score for patients over 15 years of age. Our study found a 53.8% risk of mortality among patients with a score greater than 7, which helps assess severity but it does not determine the therapy and it is not superior to clinical evaluation of the patient.

The length of stay in our unit was relatively short (median of 8 days), and this finding is probably related to the hospitalization of less severely burned patients, since the HPS is the only reference service in the state of Rio Grande do Sul for the treatment of burned children. This sample corresponds to the epidemiology of a pediatric burn treatment center but reproduces the most prevalent findings in the literature, despite the limitations found in our service.

As observed in our study, the exposure to electrical flow is not a frequent cause of burns, but it is related to greater severity, longer hospital stays and higher mortality.

We consider this study of extreme importance because it identifies factors and risk behaviors associated with the worst outcomes in burned children. Based on this, it is possible to intervene through prevention policies, with which up to 70% of deaths associated with burns could be avoided.^(20)^

## CONCLUSION

Burns from scalding are quite frequent and are associated with high morbidity. However, special emphasis should be given to accidents involving fire due to the resulting high mortality rate. In these patients, it is essential to highlight diagnosis and treatment targeting the adequate management of inhalation injury.
